# Identifying and managing problematic substance use in outpatient psychiatric care: a national survey in Sweden

**DOI:** 10.1186/1940-0640-8-S1-A74

**Published:** 2013-09-04

**Authors:** Christopher Sundström, Anne H Berman, Kristina Sinadinovic

**Affiliations:** 1 Karolinska Institute, Department of Clinical Neuroscience, Center for Psychiatric Research, Stockholm, Sweden; 2 Stockholm Center for Dependency Disorders, Stockholm, Sweden

## 

About 25% of psychiatric patients are known to have problematic substance use. The National Board of Health and Welfare in Sweden recommends that treatment providers of psychiatric care systematically identify patients with problematic substance use, and provide Brief Intervention (BI) for them. It is not known to what extent these recommendations are followed. Two surveys were designed, one for directors of outpatient psychiatric clinics, the second for clinical staff at these clinics. The survey to clinic directors focused on guidelines at the clinic for identifying substance use during the assessment phase, and for offering BI to patients with problematic substance use. The survey to staff focused on identification of problematic substance use during the assessment phase and personal experiences in identifying such use as well as offering BI. The response rate for clinic directors was 60% (n=137). Most clinics had guidelines for always asking patients about substance use in the assessment phase (92% for alcohol; 80% for drugs). Guidelines existed for offering BI for hazardous alcohol use at 49%; for hazardous drug use at 42%. The response rate for staff in participating clinics was 40% (n=481), 66% stated that they always ask about alcohol use during the assessment phase. Corresponding figure for drug use was 58%. 36% reported using BI for hazardous alcohol and drug use. Guidelines for identifying problematic substance use had been formulated at almost all outpatient psychiatric clinics, but among staff, only two out of three reported applying them. Guidelines for using BI had been formulated at less than half of the clinics. Actual BI provision by staff to patients was even more rare. During 2013-14, directors and staff participating in this survey will be involved in design and implementation of e-interventions (internet self-help sites, IVR, mobile applications) for problematic substance use.

